# 4488 Neural Network of the Cognitive Model of Reading

**DOI:** 10.1017/cts.2020.415

**Published:** 2020-07-29

**Authors:** Joseph Posner, Vivian Dickens, Andrew DeMarco, Sarah Snider, Peter Turkeltaub, Rhonda Friedman

**Affiliations:** 1 Georgetown - Howard Universities

## Abstract

OBJECTIVES/GOALS: A particularly debilitating consequence of stroke is alexia, an acquired impairment in reading. Cognitive models aim to characterize how information is processed based on behavioral data. If we can concurrently characterize how neural networks process that information, we can enhance the models to reflect the neuronal interactions that drive them. METHODS/STUDY POPULATION: There will be 10 unimpaired adult readers. Two functional localizer tasks, deigned to consistently activate robust language areas, identify the regions of interest that process the cognitive reading functions (orthography, phonology, semantics). Another task, designed for this experiment, analyses the reading-related functional-connectivity between these areas by presenting words classified along the attributes of frequency, concreteness, and regularity, which utilize specific cognitive routes, and a visual control. Connectivity is analyzed during word reading overall vs. a control condition to determine overall reading-related connectivity, and while reading words that have high vs. low attribute values, to determine if cognitive processing routes bias the neural reading network connectivity. RESULTS/ANTICIPATED RESULTS: The localizer analysis is expected to result in the activation of canonical reading areas. The degree of functional connectivity observed between these regions is expected to depend on the degree to which each cognitive route is utilized to read a given word. After orthographic, phonologic, and semantic areas have been identified, the connectivity analysis should show that there is high correlation between all three types of areas during reading compared to the control condition. Then the frequency, regularity, and concreteness of the words being read should alter the reliance on the pathways between these area types. This would support the hypothesized pattern of connectivity as predicted by the cognitive reading routes. Otherwise, it will show how the neural reading network differs from the cognitive model. DISCUSSION/SIGNIFICANCE OF IMPACT: The results will determine the relationship between the cognitive reading model and the neural reading network. Cognitive models show what processes occur in the brain, but neural networks show how these processes occur. By relating these components, we obtain a more complete view of reading in the brain, which can inform future alexia treatments.

